# Information Mismatch: Cancer Risk Counseling with Diverse Underserved Patients

**DOI:** 10.1007/s10897-017-0089-4

**Published:** 2017-03-13

**Authors:** Galen Joseph, Rena J. Pasick, Dean Schillinger, Judith Luce, Claudia Guerra, Janice Ka Yan Cheng

**Affiliations:** 1 Department of Anthropology, History & Social Medicine, Helen Diller Family Comprehensive Cancer Center, University of California, 1450 3rd Street, Rm 551, San Francisco, CA 94143 USA; 2 Helen Diller Family Comprehensive Cancer Center, University of California, San Francisco, USA; 3 Department of General Internal Medicine, University of California, San Francisco, USA; 4 Department of Medicine, University of California, San Francisco, USA; 5 Department of Psychiatry, University of California, San Francisco, USA

**Keywords:** Health communication, Patient-provider communication, Hereditary cancer, Disparities, Low-income, Health literacy, Limited English proficiency (LEP), Immigrant Health

## Abstract

As genetics and genomics become part of mainstream Medicine, these advances have the potential to reduce or exacerbate health disparities. Gaps in effective communication (where all parties share the same meaning) are widely recognized as a major contributor to health disparities. The purpose of this study was to examine GC-patient communication in real time, to assess its effectiveness from the patient perspective, and then to pilot intervention strategies to improve the communication. We observed 64 English-, 35 Spanish- and 25 Chinese-speaking (*n* = 124) public hospital patients and 10 GCs in 170 GC appointments, and interviewed 49 patients who were offered testing using the audio recordings to stimulate recall and probe specific aspects of the communication. Data analyses were conducted using grounded theory methods and revealed a fundamental mismatch between the information provided by GCs and the information desired and meaningful to patients. Several components of the communication that contributed to this mismatch and often resulted in ineffective communication included: (1) too much information; (2) complex terminology and conceptually difficult presentation of information; (3) information perceived as not relevant by the patient; (4) unintentional inhibition of patient engagement and question-asking; (5) vague discussions of screening and prevention recommendations. Our findings indicate a need to transform the standard model of genetic counseling communication using evidence-based principles and strategies from other fields of Medicine. The high rates of limited health literacy in the US, increasing access of diverse populations to genetic services, and growing complexity of genetic information have created a perfect storm. If not directly addressed, this convergence is likely to exacerbate health disparities in the genomic age.

## Introduction

As genetics and genomics become part of mainstream Medicine, these advances have the potential to reduce or exacerbate health disparities. Hereditary cancer services are becoming more accessible to a broader cross-section of the US population as criteria for cancer genetic services expand, insurance coverage increases, and costs go down. Yet access alone is not sufficient to ensure high quality GC and appropriate testing. Gaps in effective communication (where all parties share the same meaning) are widely recognized as a major contributor to health disparities (US Department of Health and Human Services, Office of Disease Prevention and Health Promotion 2000; Ad Hoc Committee on Health Literacy for the Council on Scientific Affairs, American Medical Association 1999; Andrus and Roth 2002; Williams 1995).

Thirty-six percent of Americans have limited health literacy (LHL), meaning they have *below basic* (no more than the most simple and concrete literacy skills) or *basic* (skills necessary to perform simple and everyday literacy activities) literacy (Kutner et al. 2006; Nielsen-Bohlman et al. 2004). Poor health literacy (HL) is a stronger independent predictor of a person’s health than age, income, employment status, education level, and race/ethnicity (Berkman et al. 2011; Weiss et al. 2005). Patients with limited English proficiency (LEP), and the elderly, poor, less educated, Latino or African American, and those on Medicaid or Medicare are more likely to have LHL, and the combination of LHL and LEP are synergistic with respect to patients’ experiences of communication (Kutner et al. 2006; Rudd 2007; Sudore et al. 2009). Individuals with LHL are less likely to actively participate in health care decision-making discussions, and more likely to struggle with health management tasks including navigating the health care system (Martin and Parker 2011). Importantly, HL is promoted by accommodating or matching provider communication with patient communication capacities (Brach et al. 2012, 2014).

The urgency to address literacy, language and culture in genetics and genomics communication has never been greater. The National Human Genome Research Institute 2013 report on the status of genomics literacy of the US defined *genomic health literacy* (based on the IOM health literacy definition; Nielsen-Bohlman et al. 2004) as the capacity to obtain, process, understand, and use genomic information for health-related decision-making. The authors identified genomic literacy as necessary to realize the promise of Genomic Medicine (Hurle et al. 2013).

While disparities in awareness and receipt of genetic testing for Hereditary Breast and Ovarian Cancer (HBOC) have been well documented (Butrick et al. 2015; Cragun et al. 2015; Mai et al. 2014; Walcott et al. 2014), relatively little research has been conducted on the communication of genetic counselors (GCs) or other genetics professionals with underserved patients (Ellington et al. 2007; Erby et al. 2008; Lea et al. 2011; Roter et al. 2007), and the potential impact of communication on disparities in the translation of genomic medicine (Forman and Hall 2009). Notable exceptions suggest that culturally tailored communication may increase awareness of genetic risk and satisfaction among African American women (Charles et al. 2006), and communication effectiveness with Chinese Australians (Barlow-Stewart et al. 2006). Research with Latino patients in prenatal genetics identified confusion and misunderstanding due to non-directive communication, jargon, inadequate translation, patient mistrust, excess cultural sensitivity, and untested formats for presenting risk information (Browner et al. 2003; Eichmeyer et al. 2005; Penchaszadeh 2001; Rapp 1993). Studies of GC literacy indicate that the literacy demands of genetic counseling may paradoxically reduce knowledge and satisfaction; however these studies were conducted with simulated GC sessions rather than actual visits (Erby et al. 2008; Lea et al. 2011; Roter et al. 2007).

To our knowledge, ours is the first study to examine the communication between GCs and low income, ethnically and linguistically diverse patients as they undergo cancer risk counseling (Meiser et al. 2008; Paul et al. 2015). The purpose of our study was to examine GC-patient communication in real time and to assess its effectiveness from the patient perspective.

## Methods

We used multiple inductive methods, including standard ethnographic techniques to conduct systematic observations designed for minimal disruption of usual routines (Atkinson and Hammersley 1994; Denzin and Lincoln 1998; Johnson and Sackett 1998). We audio-recorded GC sessions, and conducted stimulated recall interviews with observed patients who were offered testing (Lyle 2003; O’Brien et al. 2008). Additional methods used but not reported on here, include semi-structured interviews with observed GCs and healthcare interpreters, and pilot testing of strategies to improve GC communication. All research procedures for this study were approved by appropriate Institutional Review Boards. In accordance with our IRB approved protocol, all proper names are pseudonyms, and we have changed potentially identifying characteristics to protect individuals’ identities.

### Settings

Over 30 months (November 2012 – April 2015), we conducted direct observations at two public county hospitals in large metropolitan areas of California where nearly all patients have Medicaid or Medicare or are uninsured. Masters level licensed GCs provided counseling and testing, which was available to patients free of charge through a variety of means, including Medi-Cal (California’s Medicaid program), Medicare, county health programs, laboratory hardship programs and foundation support.

At Site 1, GCs who specialize in cancer see patients independently. Patients are referred through community clinics and the hospital’s mammography, primary care, and oncology clinics. GCs typically see a patient two or three times: (1) a pre-test appointment to discuss family history, assess risk, and educate about hereditary cancer and genetic testing; (2) a second pre-test appointment to update pedigree with additional information if requested by the GC, review risk assessment and pre-test education, and draw blood/collect saliva; (3) a results appointment to discuss implications of test results for patient and family, and screening/prevention options. Some positive patients also return for a follow-up appointment. Medical interpretation is provided remotely by phone or video.

At Site 2, GCs are generalists who see patients with the support of an MD geneticist who was sometimes consulted during the visit or joined the GC for part of the visit. Patients are referred through oncology and primary care. GCs typically see patients for a pre-test appointment to assess risk and draw blood, and call the patient with test results. Patients with positive or variant of uncertain significance (VUS) results are invited for an in-person follow-up appointment. Medical interpretation is provided remotely by phone.

### Participant Eligibility and Data Collection Procedures

All English-, Spanish- and Chinese-speaking patients who had appointments when a language concordant researcher was available were eligible for inclusion in the study. Therefore, some eligible participants may not have been offered the opportunity to participate. Initially, the study focused on patients being counseled for HBOC, but as the testing environment changed with the advent of panels after the June 2013 Supreme Court decision overturning the Myriad patent (*Association for Molecular Pathology v. Myriad Genetics*), we expanded our criteria to include patients suspected of having Lynch and other hereditary cancer syndromes who were frequently being offered the same panel test. Except for the occasions when a patient declined participation or asked not to be recorded, the researcher directly observed and audio-recorded patient-genetic counselor appointments. Spanish and English speakers rarely declined to be audio recorded; in contrast, 12 of 40 observed Chinese participants did not consent to be recorded. The researcher took detailed field notes to record the dynamics of each session, communication challenges, emotional tenor, body language, etc. (Emerson et al. 1995). We obtained verbal consent from counselors and patients for all observations and written consent for all interviews.

After the initial visit, all observed patients who were offered genetic testing were invited to complete a semi-structured interview using stimulated recall (Lyle 2003; O’Brien et al. 2008). We continued to observe and conduct interviews until reaching theoretical saturation (Glaser and Strauss 1967). Interviews were conducted as soon as possible after the appointment (median = 8 days). Observation fieldnotes and audio recordings were used to tailor the interview guide which included the following general topics: (1) experience with GC and testing; (2) understanding of inheritance and beliefs about the causes of breast cancer; (3) cancer risk perceptions; (4) understanding of test results and screening recommendations; and (5) personal history and sociocultural and socioeconomic context of daily life. Audio excerpts were used in each interview for stimulated recall (unless audio recording had been declined). We selected standard elements of the GC communication such as the explanation of genes, heredity, BRCA, possible test results, and implications of test results for patient and family, as well as excerpts where patients actively participated or were non-responsive in order to probe patients’ understanding or thinking. The number of excerpts varied according to the content of the session and the patient’s stamina and patience with reviewing the audio recording (range 6–17).

At the end of each initial interview, we administered a short demographic survey and the Subjective Numeracy Scale (SNS) (Fagerlin et al. 2007; Zikmund-Fisher et al. 2007). SNS is an 8-item scale that measures self-perceived ability to perform mathematical tasks (e.g. calculating a tip) and preference for numerical rather than prose information. Each question is scored on a 6-point Likert-like scale, and the overall score is computed as the average rating across all eight questions. The SNS has been validated in English (Zikmund-Fisher et al. 2007); we used a professional translation of it for Spanish and Chinese speakers. We invited patients for a second interview after their results appointment using the same procedures. In appreciation of their time, respondents received a $25 gift card for the first interview, and a $35 gift card for the second interview.

### Data Analysis

We calculated demographic characteristics and SNS for interview participants. As a first level of analysis, prior to each qualitative interview, the interviewer reviewed observation field notes and the audio recording to identify appropriate excerpts for stimulated recall and to tailor the interview guide accordingly. Audio recordings of interviews, including the segments of observations played during the interviews, were professionally translated/transcribed, and transcripts were entered into the qualitative analysis software Atlas-ti 7 for coding. We followed standard techniques based in grounded theory, including iterative data review, and use of multiple coders to identify the themes described and illustrative quotes included below (Bernard 2006; Glaser and Strauss 1967; Strauss and Corbin 1990). Each transcript was read by three members of the research team and coded by at least two who also wrote interim analytic memos. Coders independently reviewed the initial transcripts using a combination of open coding and a priori codes based on the interview guide, the literature, and preliminary research (Joseph and Guerra 2015). Coders then met to reconcile discrepancies and establish a codebook. Examples of commonly used codes include: GC explanation of possible test results; GC explanation of screening/prevention options; patient information needs; patient understanding of genetic test; patient understanding of screening/prevention options. Subsequently, coders independently coded using the codebook, and then met to reconcile discrepancies, discuss adding new codes as needed, and to discuss coding memos which described emerging themes.

## Results

### Participants

We observed English- (64), Spanish- (35) and Chinese-speaking (25) patients (*n* = 124, plus 24 accompanying family members) during 170 GC sessions with 10 GCs at the two sites. Sessions included pre-test, results and results follow up appointments. We invited all 49 patients who were offered testing to participate in an interview, and all accepted either after the pre-test or results appointment. (See Table 1). We conducted 58 post-visit interviews with the 49 patients; 35 were conducted after a pre-test appointment; 23 after a results appointment. All but four patients who were offered genetic testing accepted the test. Nine received positive results; five received a variant of uncertain significance, and 31 tested negative.

**Table 1:** Study sample characteristics by language group

	English	Spanish	^a^Chinese	Total
Observed sessions Pre-test Results Follow up	87 68 14 5	43 31 11 1	40 29 9 2	170 128 34 8
Individual patients observed	64	35	25	124
Observed patients offered testing/declined testing	17/3	20/1	13/0	50/4
Interviewed/Interviews (including test decliners)	16/17	20/24	13/17	49/58
Interviewed after				
Pre-test	11	15	9	35
Result	6	9	8	23
Cancer status	6 breast 3 ovarian 7 unaffected	^b^11 breast 1 melanoma 8 unaffected	9 breast ^c^1 ovarian 1 colon 1 rectal 1 unaffected	^b^25 Breast (incl DCIS) ^c^3 ovarian 1 each melanoma, colon, rectal 16 unaffected
Test results				
Positive	3	3	3	9 (6 BRCA; 2 Lynch; 1 ATM)
Negative	10	16	5	31
VUS	0	0	5	5
Not tested	3	1	0	4

^a^ 37 Cantonese, 2 Mandarin, 1 Toisanese

^b^One had breast and lung

^c^One had ovarian and uterine

Demographic characteristics of the interviewed patients are in Table 2. Chi-square tests showed significant differences in educational level by language group. Of the English-speaking participants, 70.6% had some college education or above compared with 37.5% of the Spanish-speaking participants and 16.7% of the Chinese-speaking participants. A one-way ANOVA revealed that the overall Subjective Numeracy Scale (SNS) scores (average rating across all 8 items) differed significantly by language group, *F* (2, 44) = 3.28, *p* < .05 (Table 2). Tukey’s HSD (honest significant difference) tests showed that English-speaking participants had higher scores than Spanish-speaking participants, indicating that English-speaking participants perceived higher ability to perform various mathematical tasks and preferred the use of numerical information over prose. English- and Chinese-speaking participants did not significantly differ in overall SNS scores. For comparison, in the original scale development article, Fagerlin et al. (2007) reported an average score of 4.03 (*SD* = 1.04; range = 1.00 to 6.00) in a sample with higher levels of education (72% self-identified as White, 52% had some college, 10% had at least a bachelor’s degree).

**Table 2:** Demographic characteristics of interview sample by language (*N* = 49)

	English (*n* = 16)	Spanish (*n* = 20)	Chinese (*n* = 13)	Total (*n* = 49)
	Mean (*SD*) or n (%)			
^*a*^Age	49.44 (13.72)	42.80 (11.22)	55.75 (12.78)	48.25 (13.29)
Marital status				
Married/with a long-term partner	6 (37.5)	13 (65)	10 (76.9)	29 (59.2)
Never married	4 (25)	1 (5.0)	0 (0)	5 (10.2)
Legally separated or divorced	4 (25)	5 (25.0)	0 (0)	9 (18.4)
Widowed	1 (6.3)	1 (5.0)	1 (7.6)	3 (6.1)
Unreported/missing	1 (6.3)	0 (0)	2 (15.4)	3 (6.1)
Highest level of education				
Less than high school	1 (6.3)	10 (50.0)	5 (38.5)	16 (32.7)
High school or equivalent	3 (18.8)	3 (15.0)	4 (30.8)	10 (20.4)
Some college or higher	11 (68.8)	7 (35.0)	2 (15.4)	20 (40.8)
Other (vocational school)	0 (0)	0 (0)	1 (7.6)	1 (2.0)
Unreported/missing	1 (6.3)	0 (0)	1 (7.6)	2 (4.1)
^*a*^Subjective Numeracy				
Range	2.38–5.50	1.00–5.75	1.63–5.13	^*d*^ *F* = 3.28
Mean (SD)	4.23 (0.86)	3.12 (1.61)	3.66 (1.06)	
Race/ethnicity				
African American	2 (12.5)	0 (0)	0 (0)	2 (4.1)
Chinese	0 (0)	0 (0)	13 (100)	13 (26.5)
^*b*^Hispanic/Latino	1 (6.3)	19 (95.0)	0 (0)	20 (40.8)
White	11 (68.8)	1 (5)	0 (0)	12 (24.5)
^*c*^Other	2 (12.5)	0 (0)	0 (0)	2 (4.1)
U.S. born				
Yes	11 (68.8)	1 (5.0)	0 (0)	12 (24.5)
No	4 (25.0)	19 (95.0)	13 (100)	36 (73.4)
Missing	1 (6.3)	0 (0)	0 (0)	1 (2.0)
Years in the U.S. (if foreign-born; *n* = 35)				
Range	12–52	5–30	1–37	1–52
Mean	36	14.8	14.8	17.24
Language(s) Spoken at home				
Only non-English/More non-English language than English	5 (31.3)	18 (90.0)	13 (100.0)	36 (73.5)
Both equally	0 (0)	2 (10.0)	0 (0)	2 (4.1)
^*e*^Only English/more English than non-English language	11 (68.8)	0 (0)	0 (0)	11 (22.4)
Preferred Language with your doctor and nurses				
English	13 (81.3)	4 (20.0)	0 (0)	17 (34.7)
Spanish	1 (6.3)	16 (80.0)	0 (0)	17 (34.7)
Cantonese/Mandarin	0 (0)	0 (0)	13 (100.0)	13 (26.5)
English/another non-English language	2 (12.5)	0 (0)	0 (0)	2 (4.1)

^*a*^Missing *n* = 1 Chinese

^*b*^“Hispanic/Latino” included 6 Hispanics/Latinos, 9 Mexicans, 2 Nicaraguans, and 3 Salvadorans

^*c*^“Other” includes 1 Filipino and 1 White/American Indian

^*d*^ *p* < .05

^*e* “^Only English/more English than non-English language” includes 1 English/Tagalog and 1 English/Portuguese

Table 3 shows demographic characteristics of observed GCs. Observed pre-test GC appointments lasted 25–75 min with an average of 45–53 min depending on language and site. The majority of each session consisted of obtaining the family history and education about hereditary cancer and genetic testing, while counseling and psychosocial support typically constituted a minor portion of the session.

**Table 3:** Demographics of observed genetic counselors

Site	Gender	Race/Ethnicity	Years in practice (range)	Total
1	3 women	3 White	4–25 years	3
2	5 women 2 men	5 White 1 African American 1 Asian American/White	3 months - 25 years	7
Total				10

### Key Qualitative Themes

Through analysis of the observations and interviews, we identified a fundamental mismatch of patient information needs and information provided by counselors (Table 4). Here we describe several components of the communication that contributed to this mismatch, along with illustrative quotes from our patient interviews. These components often resulted in ineffective communication: (1) too much information; (2) complex terminology and conceptually difficult presentation of information; (3) information perceived as not relevant by the patient; (4) unintentional inhibition of patient engagement and question asking; (5) vague discussions of screening and prevention recommendations. Quotes are labeled with the participant’s language (ENG, SP, CH), interview number and Pre-test or Results to indicate which appointment preceded the interview.

1. Too much Information
  A key component of the mismatch we identified was in the amount of information patients wanted compared with what GCs provided. While GCs are trained to provide education about genetics, we found that in the context of long explanations of heredity, genetics and genetic testing, counselors’ key messages often got lost and patients felt overwhelmed. In the following example, the GC offered “background” information that involved technical terminology (genes, cells), an analogy (“a gene is like an instruction book”), and complex scientific concepts (cell growth, death and regulation, tumor versus normal cells).

**Table 4:**
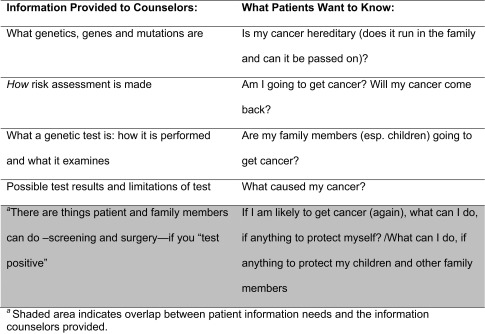
Mismatch of information provided by genetic counselors and desired by patients

*(Audio Playback of Counseling Session during Interview)*

GC: We have our genetic material in our body and we have, we actually have two copies of every gene, a gene being an instruction that keeps our body working properly, the cells working properly. When they need to, they make new cells or when they need to die, they die. So, cells are normally dying and growing at one time. So there are certain genes that regulate that process, that make sure that the cells grow and die at the right time and they don't form tumor cells.

*(Interview)*

Interviewer: What did you understand about that?

Participant: I just understood about the genes, that we only have two genes and how some eventually die and how one can still function with one. That's all that I really understood. [ENG/SP-26; GC conducted in English/Interview in Spanish; Pre-test].

Rather than clarifying, the background information confused the patient and obscured the main point that the GC wanted to convey about how cancer may be inherited.

Patients also indicated that information overload during GC not only undermined understanding, but also could impede decision-making. In the following case, this bilingual 40 year old with breast cancer was too overwhelmed to decide about having the test. She ultimately agreed after significant encouragement by the GC.

*(Audio Playback of Counseling Session during Interview)*

GC: …so if it's something that you're interested in, we could get it started today. But if it's something that you don't know if you would be interested in, you could take time to think about it. Okay?

Patient: I like [to] take time to think about it.

GC: Okay, you're not sure if you want the test, the blood test?

Patient: Mm-hm.

GC: Okay. Tell me more. Tell me what--what are your thoughts?

Patient: The only problem is I don't like--I don't like the needles, so I just been like two times yesterday, today. So, I don't want no more today.

GC: Okay.... how important is this kind of information to you, though? Like to know if there is a genetic reason why you have cancer?

*(Interview)*

Interviewer: What were you thinking there when she asked you that?

Patient: Well, I didn't know how important because the truth is-- all of this was just like-- Ah! Too much information sometimes. So really... I knew and I know that it is important, but at that moment it was like, this is too much right now. Let me go home. [ENG/SP-65 Pre-test].

2. Complex Terminology and Conceptually Difficult Presentation of Information
  The complex terminology used by GCs and the use of conceptually complex linguistic constructions like analogies and hypotheticals contributed to patients’ misunderstanding and overwhelm. GCs used many specialized terms, including “genes”, “hereditary”, “BRCA”, “chromosome”, “deleterious”, “genetic factors”, “predisposition”, “risk”, “sporadic”, “mutation”, and “variant”. Even “genetic counselor” was a confusing term for many who had never heard it prior to being referred. “Mutation” presented particular challenges. Although counselors described “mutation” as a “change in a gene,” and then used the term interchangeably with “change,” many patients did not understand it. In the context of breast cancer treatments in which mastectomy can easily be perceived as a form of mutilation, several Spanish speakers confused the less familiar “mutation” with the more familiar “mutilation” (*mutación* vs. *mutilación*). This 62 year-old bilingual Latina struggled with the technical terminology.
  I really didn't pick up too much. I just feel that sometimes doctors go in and they're using all these words and stuff. No. Tell me layman’s terms, because I'm not dumb, but something like that I don't really understand. [28-ENG/SP Pre-test]

In her interview, it was evident that this patient understood only the most basic information the GC had provided, and was only interested in knowing if her children would inherit her cancer.

To explain the limitations of testing an unaffected individual who was referred due to family history, counselors often used hypothetical scenarios to describe why another relative might be a “more informative” person to test. In the following case, this 28 year-old patient’s mother had died of breast cancer in their home country of Nicaragua when the patient was a baby. (“(*Interpreter)*” indicates a point in the conversation where the GC paused to allow the medical interpreter to translate the counselor’s speech into Spanish).

*Audio Recording:*

GC: …The best way for us to figure out if it’s genetic would have been to actually test your mom and look at her two BRCA genes (*Interpreter*) and that would tell us, does she have a change in those genes that had contributed to her breast cancer? But because she is not alive that’s not a test we can obviously do. *(Interpreter)* So what we will do is we will go ahead and look at your BRCA1 and BRCA2 genes. *(Interpreter)* If we see a mutation then we would just make the assumption that you had inherited that mutation from your mother. *(Interpreter)* So, if your test comes back normal, meaning both genes are just the way they're supposed to be, there's no mutation, that's good news. But we still, interpret that with a little bit of caution. *(Interpreter)* …let's say your mom did have a mutation on one of these genes, let's say BRCA1 gene, we have two copies of every gene, because we get one from our mother and one from our father. *(Interpreter)* So if your mother carried a copy of this gene with a mutation, every time she'd have a child, it's a fifty-fifty chance whether she passed on the copy that causes the higher risk of cancer, or the normal copy... *(Interpreter).*

*Interview:*

Participant’s Boyfriend: Did you understand?

Participant: That I could or could not have [cancer].

Participant’s Boyfriend: Yes. It's fifty-fifty. 50% that you have cancer, or 50% that you don't. But actually the, the test they did on you of the RCA1 and RCA2 means that because of that you won't get cancer, but you could get it for some other reason. Fifty percent for some other reason. [SP-20 Results].

While the counselor intended to convey the limitations of testing an unaffected individual, the use of the hypothetical with attendant conditional tense and abstract possible future scenarios was confusing. The patient’s boyfriend struggled to understand what the 50/50 risk signified. The patient, fearing that she already had cancer, and with little formal education, had no context to make sense of the explanation of genetics and believed the test was diagnostic. Her main concern, as expressed in her interview, was not addressed in a way she understood: “I want to know if I’m going to die or not.”

While working through a medical interpreter could amplify the difficulty of such conversations, this complexity could be confusing for English speakers with more education too, like this 28 year old, college educated woman whose mother had died of ovarian cancer.

*Audio Recording*

GC: So it’s true that the likelihood is that -- you know, almost nine out of ten ovarian cancers are not genetic. They happen just by chance. And that would certainly be the most likely given that there’s no other cancers, but [your mother’s] family is kind of relatively small. So we do …offer genetic testing to any individual who’s had ovarian cancer even if there’s no family history just because of that kind of 5 to 10% chance that it would be hereditary. And the guidelines also kind of say that it’s appropriate to offer testing to an offspring of somebody who’s had ovarian cancer if they’re no longer willing to test.

*Interview*

Interviewer: Yes, there’s kind of a lot there. What’s your reaction to all that or any of that?

Patient: Yeah, just getting more information, I suppose. I still am like a little unclear. Like I remember feeling this way in appointments and just kind of like being lost in like a little bit of like what sounded like medical jargon, you know, like stuff that I just – you know, if I hear a word that I don’t recognize, I’m kind of like lost in the meaning of the sentence, I suppose. But also just, yeah, gathering information about what the whole process is about and how they select their candidates. [ENG-11 Pre-test].

In this case, the counselor’s attempt to explain her reasoning for offering the test, and emphasis on why the patient met testing criteria distracted the patient and made it difficult to focus on why the test might be useful for her.

In attempts to make abstract concepts more concrete, GCs frequently used analogies (e.g., “sequencing as a spellcheck”, “genes like an instruction book”, “flipping a coin”). While some patients could relate to and understand these analogies, for others lack of cultural or linguistic equivalence made them meaningless. In the following example, the medical interpreter anticipated the potential for confusion on the part of a Spanish-speaking patient.

GC: The test they do, it's very much like a spell check when you are writing a document on the computer or maybe sending a text on your phone, it underlines the words that are misspelled.

Interpreter: I don't know if you saw the computer, if you use a computer, when you write something, the computer corrects you if you write incorrectly. I don't know if you have seen that.

Patient: No.

Interpreter: Okay. Well, what she is saying is that this is a test in which later you can see on a screen like when the computer corrects your misspellings.

Patient: Yes.

Interpreter: She has not seen that on the computer.

GC: Okay. But like on your phone, if you are texting somebody...

Interpreter: But if you are sending a text message on the telephone, I don't know if you do that either, but if you are writing it corrects you automatically. I don't know if you have seen that.

Patient: I never send texts.

Interpreter: She never sends texts.

Patient: I am far from civilization.

Interpreter: I am far away from civilization.

GC: Okay, all right. Well, basically this test it goes through these genes and makes sure they're spelled correctly. [SP-29 Pre-test GC session].

Although the interpreter tried to guide her, the GC continued to try to use the spellcheck analogy. This patient never returned to the clinic after this appointment, and never responded to our attempts to reach her for an interview.

3. Information Perceived as not Relevant by the Patient

As the previous two themes suggest, patients wanted information that was simpler, narrower in scope, and more directly relevant to their immediate situation than what GCs provided (Table 4). Most importantly, they wanted information that they could not only understand, but that was relevant to their personal context. For example, understanding chromosomes was simply not important to this 62-year-old retired flight attendant recently diagnosed with breast cancer.

And chromosomes. Those were really kind of fascinating but it wasn't something that I really wanted to see. I just wanted to know how it [the test] was going to benefit me… I think the bottom line is, I just want to know at what percentage rate is it going to come back. [ENG-33 Pre-test].

Importantly, this patient did not understand that the main purpose of the test was to determine her risk for new cancers of the breast and the ovary rather than risk of recurrence. Similarly, a 46-year-old monolingual Cantonese speaker with breast cancer said she only wanted to hear about the test results. She wasn’t interested in how the test works, what it examines or how the GC makes her risk assessment. As a result, that information did not “stick”.

It wasn’t because of the interpreter, nor was it because the topic was too complicated. It mainly depends on if I think the information…has any impact on me. It’s like when I take a test I only want to know the score. I don’t care about anything else, plus I don’t even remember those other things. …Anything else just doesn’t stick in my mind. [CHI-16 Results].

The details about genetics were beyond her comprehension and interest, and therefore not relevant to her treatment or prevention decision-making. When asked later in the interview about her understanding of “mutation,” she replied:

I only wanted to know the results. Yeah, I mean I don’t understand these kinds of things. I don’t know what they do inside. I can’t change that fact…I don’t know anything about how genes mutate. They are inside our bodies. I can’t control them and I don’t know what I can do. There’s nothing I can do. [CHI-16 Results].

Again, the details about mutations and genes were of no interest to her, given that they were beyond her understanding and control.

4. Unintentional Inhibition of Patient Engagement and Question-Asking
  Despite not understanding much of what was said in their GC appointments, many patients did not ask substantive questions, and counselors often were unable to bridge this gap. While counselors frequently asked if patients had any questions, or if they were following the explanation, these patients typically said they had no questions or that they understood. For this bilingual Latina, the GC’s pace and use of technical language led her to believe she would not understand even if she asked a question.

Patient: …Another thing, she can speak fast in English, but how does she make sure we understood? Even if she asks [if we understand], most likely we're going to say “yes”. So the term genetics, medical terminology sometimes a person with less knowledge isn't going to understand anything…my daughter and I have gone to school and have a certain level of education …So, this is what I can tell you really happened that day. Because I'm even listening to it right now and I don't understand.

Interviewer: What was the reason that you didn't ask her to, for example, repeat the information?

Patient: I didn't ask her to repeat the information because even if she were to repeat it, I probably wouldn't have understood again for the same reason because there is vocabulary that's not in my, in my chip. (LAUGHS) [SP-4 Pre-test].

Other patients did not ask questions because they did not know what to ask or how to formulate the question. During our interview with this Cantonese-speaking 70 year-old, she wondered why she got breast cancer in the absence of a genetic mutation. Although the GC asked if she had any questions, the patient had declined to ask because she did not know how she was “supposed” to engage with a provider.

Patient: They said that I didn’t particularly have a greater chance than other people in getting cancer. The fact that I did get cancer, is it because of my mood, the food that I eat, the air that I breathe, or other factors? If that’s true, then everyone has a chance of getting cancer.

Interviewer: Did you ask [the genetic counselor] this question last time?

Patient: No. I didn’t know how to ask. Sometimes when I’m asked if I have any questions, I do not know what questions I’m supposed to ask. [CHI-11 Results]

Occasionally, when patients did ask questions, we sometimes observed that counselors provided indirect answers or attempted to explain the context, rather than answering directly and clearly, as in the case of this ATM carrier.

*Audio Recording*

Patient: Do you think, in my lifetime, what's the chance that I'm going to--

GC: --Develop breast cancer? That's a great question.

Patient: Yes.

GC: So, twelve or thirteen percent is the average woman. The one that you don't have that we were really worried about was like the eighty, eighty-five percent lifetime risk. For ATM carriers like you, it's about twenty-five to thirty-five percent.

*Interview*

Interviewer: So, what does it mean the percentage that she gave you, between twenty-five and thirty-five? What does that number mean to you, to you?

Patient: Means that I have thirty-five percent of chance, twenty-five, thirty-five percent to be in this case on the, the pool, the gene pool of cancer. You know. That's what I understood. She, she, she spoke very technically, you know?

Interviewer: Mm-hm.

Patient: Like, if someone been trained. But I've not been trained for that. So, yeah. She's supposed to be, be very plain, simple. Of course facts, but more the language that I understand. Not the language, you know. [ENG-59 Results].

Although the counselor’s response was clearly intended to contextualize the patient’s risk as between average population risk and high risk, the patient, who spoke English as a second language, found the explanation too technical.

5. Vague Discussions of Screening and Prevention Recommendations
  In contrast to much of the counseling content described above, GCs and patients found more common ground on the topic of risk reduction through screening and prevention options for the patient and her family. Yet, our interviews revealed that many patients left their results appointments with no clear understanding of what to do next. For example, GCs frequently used general terminology such as “breast exams” which patients understood as mammography, rather than the complete range of exams including a clinical breast exam, mammography and MRI that the GCs intended to convey. Patients often forgot or did not completely understand recommendations for the age to start screening and the specific type of screening for patients and their relatives.
  One patient who had been diagnosed with Lynch syndrome did not understand the nature of the syndrome or the difference between risk of metastasis and the risk of new primary cancers; thus she saw no reason to undergo a hysterectomy.

Patient: Right. She recommended that I remove my uterus, but I didn’t want to.

Interviewer: Why do you think she recommended you to remove it?

Patient: Well if I remove it, then [the cancer] wouldn’t go there. I didn’t think it was necessary, since it can go anywhere. If it doesn’t go to the uterus, it will go to the ovaries and my ovaries are in the same area. I don’t think it’s necessary. [9-CHI Results].

Other participants who did understand the recommendation were not confident that they would be able to obtain the recommended care. One 30-year-old unaffected BRCA+ patient explained how she felt about the counselor’s recommendations after her results appointment:

Walking away from the appointment, I was definitely feeling overwhelmed and just the idea that I should find a doctor who knows me and like gets to know me and I can have this relationship with over these years. …I was kind of thrown off by this idea of getting regular screenings…I like the idea of doing that but it felt like intimidating or kind of impossible or that I would really need to change my lifestyle to benefit my health in that way. … Realistically I’ll probably just do it every year. [ENG-17 Results].

She recognized that the recommended follow-up care, including screening by alternating MRI and mammogram every six months, presumed consistent access to health care and an ongoing relationship with a provider, but she had never had either. As a result, she was resigned to annual screening, rather than the recommended six-month interval.

## Discussion

This study of cancer risk counseling communication with diverse public hospital patients documented a mismatch between the information provided by counselors and what patients want and need to know. We found that patients preferred less information overall than counselors typically delivered, and they wanted it in a form that was less conceptually and linguistically complex. The quantity and form of information delivery unintentionally inhibited patient engagement and question asking. As a result, patients’ fundamental questions were sometimes left unanswered despite the counselors’ best intentions. These findings reflect the experiences of English-, Spanish- and Chinese-speaking participants in our study, even though English speakers had a higher educational level overall compared with Spanish and Chinese speakers and higher SNS than the Spanish speakers in our study.

The information mismatch suggests that counselors and their diverse low-income patients are operating under different assumptions when they meet in a counseling session. GCs are trained and expected to provide genetics education to facilitate informed decision-making about testing and risk reduction (Doyle et al. 2016). In accord with their training, GCs assumed that that they should explain *how* they make the risk assessment, the underlying biology of cancer heredity, and the technical process of genetic testing (Riley et al. 2012). These assumptions reflect biomedical culture with its attendant excitement about and immersion in the intricacies of genetic science and the evolving, nuanced process of risk assessment (Good et al. 2005; Weil 2000). In contrast, we found that many patients were not interested in this level of detailed education or the counselors’ underlying reasoning for their risk assessment. Without interest, or the expectation that they would understand the underlying science, they rarely asked for clarification even when they did not comprehend what the counselor was saying. Some assumed that the test would tell them if they had cancer or the likelihood of a recurrence. Some also seemed surprised when asked to make a decision about testing. After a long discussion of family history and genetics, and without a full discussion of the potential risks of testing, patients often assumed they would be tested rather than asked if they wanted the test. This unexpected insertion of non-directive communication could be confusing toward the end of a long appointment. The mismatched assumptions were not addressed adequately in the “contracting” portion of the session, and thus contributed to the mismatch of information.

As a result of the mismatch of information and assumptions, patients often left counseling without a clear understanding of the purpose and value of genetic testing or the recommended risk-reduction practices. Under these circumstances, truly informed consent for testing could not always be achieved, and importantly, the psychosocial and decision-making benefits of GC (Daly et al. 2016) eluded some of these patients. Given the contribution of ineffective communication to health disparities (Berkman et al. 2011; USDHHS, ODPHP 2000), our findings raise concerns that disparities documented in other aspects of hereditary cancer services (Cragun et al. 2015; Levy et al. 2011; Mai et al. 2014) may be perpetuated by these communication barriers.

Despite the client-centered model of GC, prior research has documented the high “oral literacy demand” of GC (Roter et al. 2007), and the dominance of informational and biomedical talk and clinician dialogue (Meiser et al. 2008; Paul et al. 2015). Our data are consistent with these findings, but furthermore indicate that patients’ poor understanding and limited engagement result from specific ways counselors present information, including the quantity of information, the lack of context and relevance, and the conceptually difficult and sometimes socio-culturally inappropriate hypothetical scenarios and analogies. Patients’ inability to recall risk assessments and screening recommendations is also consistent with prior studies (Heshka et al. 2008); however, our data elucidate the initial lack of understanding that limits recall.

Researchers and practitioners have begun to explore new models of genetic counseling due to the shortage of genetic counselors, the increasing demand for their services, and the growing complexity of testing brought about by next generation sequencing (Bradbury et al. 2015; Hooker et al. 2014). Some have called for a shift away from a “teaching model of counseling” and toward a “psychosocial model” that allows for more time counseling and less time educating (Biesecker 2016; Meiser et al. 2008). Our study suggests that to meet the needs of the increasingly diverse patients who now have access to genetic counseling and testing, any new model should incorporate the principles and strategies for effective communication with LHL individuals developed and tested in other areas of Medicine (Brega et al. 2015; USDHHS, ODPHP 2010).

### Limitations

This study has limitations. It was conducted at only two public hospitals in one state with a relatively small sample of counselors. As a result, the communication barriers we documented may be influenced by the practices of the participating counselors and the cultures of the two institutions where they worked. Nevertheless, the consistency in the content of the counseling sessions and in the responses of the diverse patient participants led us to identify strong patterns. Furthermore, the counselor sample included graduates of five different GC training programs who were both longtime practitioners and relatively new on the job. The presence of researchers during the counseling sessions may have influenced counseling dynamics in ways that we cannot know. Our interviews took place before patients received any mailed follow-up letters, which might aid understanding and/or recollection of key content from the GC session. Although follow-up patient letters potentially could mitigate some of the communication barriers we identified, the timing and content of these letters varied across providers, over the course of the study, and were not sent in all languages.

Our study primarily focused on documenting current communication dynamics and patients’ understanding and experience. GC perspectives on working with diverse, LEP and LHL patients also are critical for understanding the communication that we documented between public hospital patients and genetic counselors. For example, while several of the genetic counselors in our study acknowledged that they sometimes wondered if their patients understood all the information that they tried to convey, they also admitted not knowing how to bridge the communication gap or how to confirm the patient’s understanding when they suspected a problem (unpublished data). A full examination of the perspectives of the GCs in our study is beyond the scope of this paper, but will be reported in a subsequent publication. Cultural issues specific to Latina and Chinese immigrant patients, and communication challenges specific to medical interpretation also will be described in subsequent publications.

### Implications for Practice

Expanding testing criteria, health care reform, and reduced costs are making counseling and testing available to many more patients of LHL, LEP, and diverse cultural backgrounds. Going forward, the All of Us Research Program (formerly known as the Precision Medicine Initiative) and other NIH initiatives that require inclusion of diverse populations in genomic research will expose broad segments of the US population to genomic medicine. Although inequities in utilization of cancer genetic testing persist (Armstrong 2005; Levy et al. 2011; McCarthy et al. 2016; Olaya et al. 2009; Pal et al. 2014), studies show that there is interest in genetic services among such diverse populations (Komenaka et al. 2016; Ramirez et al. 2015; Ricker et al. 2006).

Given the findings of numerous studies regarding the literacy demand of genetic counseling and poor retention of information conveyed during genetic counseling (Roter et al. 2007; Meiser et al. 2008; Paul et al. 2015), it is worth considering the extent to which our findings might be relevant for all patients, not just those of limited literacy. Only 12% of the US population has proficient health literacy, meaning they can complete tasks such as calculating an employee’s share of health insurance costs using a table (Kutner et al. 2006; Nielsen-Bohlman et al. 2004). Furthermore, health information and the healthcare system can be difficult for highly skilled people for a variety of reasons, including: the complexity of information presentation; use of unfamiliar scientific and medical jargon; demands of navigating the healthcare system, such as locating providers and services and filling out forms. Perhaps most important for the genetic counseling setting, people of all literacy levels have difficulty understanding information when facing a new diagnosis or a stressful medical situation (Kutner et al. 2006; USDHHS 2008). As such, the Agency for Healthcare Research and Quality (AHRQ) suggests using a “Health Literacy Universal Precautions Approach” to making health information and healthcare contexts accessible for everyone (AHRQ 2015; DeWalt et al. 2011). This approach incorporates the principle of using plain language. As Stableford and Mettger (2007) explain, the use of plain language, which also has been endorsed by international bodies (WHO, European Commission), is “not about “dumbing down” information, writing in a condescending tone, or neglecting the need for accuracy” (p.79). Rather, it is about clarity and meaning in written and oral communication.

The science of health communication has identified additional communication principles that are relevant for the practice of genetic counseling (Coleman 2011). First, the responsibility for effective communication belongs to the clinician, not the patient; it is the clinician’s responsibility to meet the patient at his/her literacy level. Second, patients may provide cues to their literacy level, and clinicians should be trained to recognize these cues and adjust accordingly. However, since it is not always possible to tell an individual’s literacy level, the Universal Precautions Approach should be used (AHRQ 2015). Third, adapting appropriately for limited literacy requires commitment, sensitivity, flexibility, and *practice.* Finally, patient comprehension can and must be verified (Coleman 2011; Schillinger et al. 2003; Sudore and Schillinger 2009). Evidence-based communication strategies using these principles include limiting information to what the patient needs to know and needs to do; using plain language; using proven risk communication strategies (e.g. pictograms rather than percentages); and employing “teach-back” to assess patient comprehension in order to immediately adjust message and terminology (Brega et al. 2015; Fagerlin et al. 2011; Nouri and Rudd 2015; Schillinger et al. 2003; Weiss 2007).

Our group has taken a three-pronged approach that acknowledges the roles of the counselor, patient and medical interpreter to improve cancer risk communication with diverse patients across literacy, language and culture. We have developed (and are in the process of updating) tailored pre-counseling educational materials to support patients coming to genetic counseling with little or no prior knowledge of counseling or testing (Joseph et al. 2010). In addition, we have developed a training curriculum for healthcare interpreters in cancer genetics (Lara-Otero et al. 2016; Roat et al. 2016) to support interpreters’ continuing education in the field of genetics. To aid counselors, we have begun to adapt to the cancer GC context the evidence based communication strategies described above and to test the feasibility of their implementation.

## Conclusion

Our findings indicate a need to transform the standard model of genetic counseling communication to adapt to the communication needs of patients. The high rates of LHL in the US, increasing access of diverse populations to genetic services, and growing complexity of genetic information have created a perfect storm. If not directly addressed, this convergence is likely to exacerbate health disparities in the genomic age.
